# Exploring the Biological Width in Dentistry: A Comprehensive Narrative Review

**DOI:** 10.7759/cureus.42080

**Published:** 2023-07-18

**Authors:** Sayem A Mulla, Amit Patil, Sheetal Mali, Ashish Jain, Deepak Sharma, Himmat C Jaiswal, Hrishikesh A Saoji, Ashima Jakhar, Shefali Talekar, Shruti Singh

**Affiliations:** 1 Dentistry, Bharati Vidyapeeth (Deemed to be University) Dental College and Hospital, Navi Mumbai, IND; 2 Conservative Dentistry and Endodontics, Bharati Vidyapeeth (Deemed to be University) Dental College and Hospital, Navi Mumbai, IND

**Keywords:** surgical crown lengthening, periodontium, periodontal health, forced tooth eruption, biological width

## Abstract

Biological width (BW) is the distance established from the junctional epithelium and connective tissue attachment to the root surface of a tooth. It acts as a natural seal protecting the tooth from infections and diseases. The normal dimension of it is 2.04 mm on average. A periodontal probe is used to determine BW in routine clinical practice. Various methods are available for the determination of BW. A diagnosis of BW violation is asserted when the distance is found to be less than 2 mm at single or multiple locations. Gingival health is of utmost importance when considering the long-term health of the tooth as well as any restoration. A plethora of BW violations can lead to a myriad of complications, which are discussed briefly in this article. The article also aims to highlight BW in relation to restorative margins and implants and its clinical assessment as well as shed light on the procedure that can be employed to correct BW violations in dental practice.

## Introduction and background

What is biological width?

The human body is vulnerable to invasion by a variety of bacteria, pathogens, and foreign particles. Tissue derived from the ectoderm plays an important role in protecting against these disease-causing entities. The biological width (BW) that is derived from the ectoderm refers to the innate protective barrier that forms around the alveolus, protecting it from infections and diseases [1].

Over the years, different authors have defined the BW differently (Table 1) [2-7].

**Table 1:** Definitions of biological width by different authors

Authors	Year	Definition	Reference
Ingber J et al. & Amiri-Jezeh M et al.	1977 & 2006	"The junctional epithelium and supracrestal connective tissue attachment surrounding every tooth."	^[2,3]^
Nevin et al.	1984	"The sum of the combined supracrestal fibers, the junctional epithelium, and the sulcus."	^[4]^
Khuller N et al. & Nugala B et al. (most accepted)	2009 & 2012	"The dimension of the soft tissue, which is attached to the portion of the tooth coronal to the crest of the alveolar bone."	^[5,6]^
World Workshop on the Classification of Periodontal and Peri-Implant Disease and Conditions	2018	"Commonly used clinical term to describe the apico-coronal variable dimensions of the supracrestal attached tissues."	^[7]^

## Review

History of biological width

**Figure 1:**
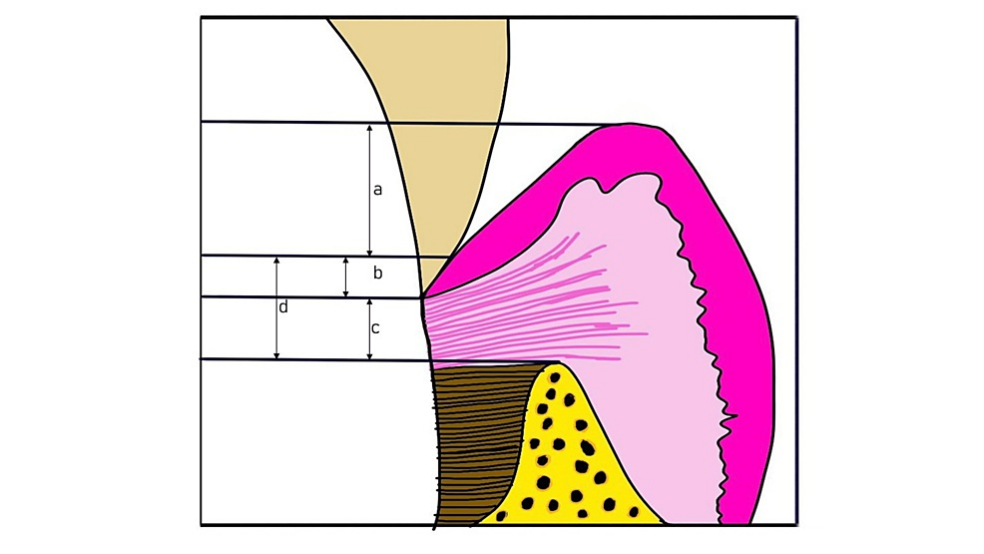
(a) Histological sulcus of depth 0.69 mm, (b) Epithelial attachment of 0.97 mm, (c) Connective tissue attachment of 1.07 mm, & (d) Biological width (b+c) Adapted from [9,10]

The term BW was first coined by Walter Cohen and described by Ingber et al. in 1977 [2]. The dimensions as well as the relationship of the dentogingival junction (DGJ) in humans were described by Gargiulo et al. Measurements of four different dentogingival components, viz. the alveolar crest, the attachment of the connective tissue, the epithelial, and the depth of the sulcus were recorded, which revealed a definite inter-proportional relation. According to their findings, the average dimensions include a mean histological depth of sulcus of 0.69 mm with an attachment of epithelial (junctional epithelium) of 0.97 mm (0.71-1.35 mm) and a supra-alveolar attachment of connective tissue of 1.07 mm (1.06-1.08 mm) (Figure 1) [8]. Hence, the overall width of the attachment is 2.04 mm (1.77 to 2.43 mm), which is referred to as the biological width and is necessary for maintaining periodontal health and removing the peeve that could harm the periodontal area such as prosthetic restorations [9,10].

Importance or significance of biological width

BW encroachment becomes a major concerning factor when there is an indication of the restoration of a tooth that has been fractured or has deep caries near the alveolar crest area [5]. The ectodermal tissue present in the body acts as a protective barrier against the invasion of bacteria and other foreign particles. Yet, this protective barrier must be crossed by both tooth and dental implants. The biological width is the term for the organic seal that forms around both, defending the alveolar bone from illness and infection.

A dentist carrying out restorations must avoid disturbing the junctional epithelium or connective tissue apparatus while preparing and recording the impression in case of subgingival margins. Since it is unfeasible for a dental clinician to accurately identify where the junctional epithelium ends and the sulcular epithelium begins, it is always advised to keep the subgingival margin extension around 0.5-1.0 mm from the gingival level [4]. In natural dentition, the shape and form of the teeth are partially correlated with gingival morphology. Different tooth shapes include triangular, ovoid, and square, as well as long narrow and short wide [11]. A triangular tooth form depicts a proximal contact to the concerned tooth that is positioned more toward the incisal aspect and requires a larger quantity of tissue height to deputize, which puts it at a perilous of developing the "black hole disease," whereas people with square teeth have longer proximal contacts and less papillary tissue [12].

Dimensions of periodontium

The periodontium was categorized into three dimensions by Maynard and Wilson [13], superficial physiologic gingiva that surrounds the tooth in its free and connected state. Physiologically, the crevicular dimension is the distance between the junctional epithelium and the gingival margin. Subcrevicular physiologic refers to the biological width and is made up of the connective tissue connection and junctional epithelium.

Clinical evaluation of biological width

The determination of BW in clinics is done using a periodontal probe. A diagnosis of violation of BW is asserted when the distance is found to be less than 2 mm at single or multiple locations. Measurements should be taken on more than one tooth with healthy gingiva to diminish the chances of site and individual variations, thus sealing precise gauging [6,14].

Methods of clinical evaluation of biological width

Clinical Method

A solid indication of biological width violation due to the extension of restoration margins into the attachment is when a patient complains of soreness around the tissues when the levels of restoration margin are being assessed with a periodontal probe. The following symptoms of biological width violation include clinical attachment loss, alveolar bone loss, gingival recession, pocket formation, chronic form of inflammation of the gingiva around the restored tooth, gingival hyperplasia, which is localized in a location with the minimum amount of bone loss and bleeding when probed. The most frequent locations for gingival hyperplasia are passive eruption, which is usually altered, and restorative borders placed below the gingiva [15].

Bone Sounding

BW can be determined by "sounding to bone" (probing to the bone level while under local anesthetic) and deducting the sulcus depth from the resultant measurement. BW violation can be diagnosed if this distance comes out to be less than 2 mm at single or multiple places. To obtain an accurate assessment and minimize individual and site variability, this measurement should be carried out on multiple teeth with healthy physiologic periodontium.

Radiographic Method

The interpretation of radiographs can spot interproximal breaches of biological width. However, due to dental superimposition, radiographic techniques are not expository on the mesiofacial as well as distofacial line angles of teeth [16]. To determine the size of the DGU, a unique parallel profile radiography (PPR) technique has been described. Given that the PPR methodology was straightforward, succinct, non-invasive, and repeatable, the authors deduce that it could be used to precisely quantify the length and thickness of the DGU [17].

Categories of biological width

**Figure 2:**
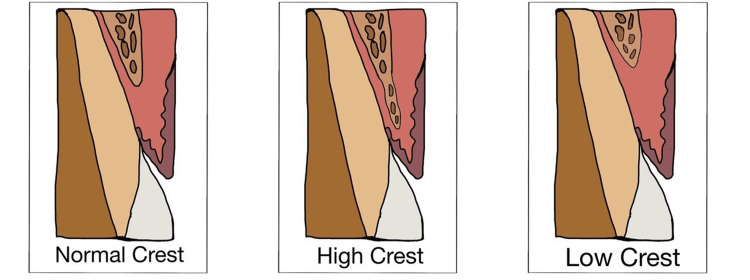
Categories of biological width Adapted from [7]

Following bone-sounding measures, Kois proposed three types of BW in patients [17]. Patients with normal crests, low crests, and high crests are among the classifications (Figure 2).

Normal Crest Patients

These are seen in 85% of cases The proximal measurement ranges from 3.0 mm to 4.5 mm while the mid-facial measurement is 3.0 mm in patients with normal crest. In these situations, the gingival tissue frequently exhibits long-term stability. A crown's edge shouldn't be situated any closer to the alveolar bone than 2.5 mm. As a result, in patients with normal crest, a crown margin that is positioned 0.5 mm subgingivally has a tendency to be well endured by the gingival tissues, thus attaining stability over time.

Low Crest Patients

These are seen in 13% of cases. The proximal measurement is greater than 4.5 mm and the mid-facial measurement is larger than 3.0 mm in patients belonging to the low crest group. Because an intracrevicular crown margin was used, the patients depicting low crest have traditionally been thought to be supplemental and prone to recession. The attachment apparatus frequently suffers damage when a retraction cord is inserted during crown preparation. A gingival recession happens as a result of the wounded attachment's tendency to repair back to its normal crest position as it recovers. Because not every low crest patient responds the same way to an attachment injury, an attachment at the lower crest is actually more complicated. While some patients with low crest have an attachment system that is relatively secure, others are prone to gingival recession. The depth of the sulcus is the differentiating factor that can have a wider range.

High Crest Patients

These are seen in 2% of cases. This is more frequently observed on a proximal surface near an edentulous spot. The mid-facial measurement and proximal measurement in the high crest patient are both less than 3.0 mm. The margin would be excessively close to the alveolar bone in this case, leading to biological width impingement, and persistent inflammation would likely arise from placing an intracrevicular margin.

Importance of determining the crest category of the patient

Clinicians must have a thorough understanding of the crest category while preparing anterior teeth for indirect restorations. The clinicians can choose an ideal location for the margin by determining the crest category. It is viable to place an intra-crevicular margin with the goal of long-term stability and aesthetics [2,16-18].

Certain guidelines can be employed to position intracrevicular boundaries based on the sulcus depth. First, the margins of the restoration can be positioned 0.5 mm below the gingival tissue crest if the probing depth of the sulcus is 1.5 mm or less. Second, the margins of the restoration should be inserted in the sulcus at a depth that is half its probing depth if it is deeper than 1.5 mm. Lastly, a gingivectomy may be enacted to extend the tooth and design a 1.5 mm sulcus if the probing depth of the sulcus is greater than 2 mm [19,20].

Biological width around implants

Two-piece implants have a wider biological width than single-piece implants and natural teeth. The presence of a microgap and where it is located affect the biological width of the surrounding soft tissue and the marginal bone levels. The connective tissue surrounding implants is more stable than the epithelial dimension [21]. The development of biological width is a physiological reaction in the mouth cavity and is independent of loading quantity or quality [22]. Connective tissue dimensions are more stable around one-piece implants and natural teeth. However, microbial development and pathologic microbial products present a continual threat to the junctional epithelium. Independent of tissue biotype (thick/thin), the biological width that has invaded the area around the implant experiences identical structural and histologic changes to those that are visible around the tooth [23].

Significance of biological width and margin placement: two parts of the same coin

Margin placement can be done at three different levels: supragingival, subgingival, and subgingival.

Supragingival Margins

It affects the periodontium the least. Due to the pronounced incongruity in color and opacity of conventional restorative materials against the tooth, this border has been used in non-esthetic locations. Advantages like ease, simplicity, and least irritation to gingiva are achieved [3].

Equigingival Margin

Equigingival edges were believed to induce higher plaque buildup and gingival irritation than supragingival or subgingival margins. Today, however, it is possible to aesthetically merge the restorative margins with the tooth and design them to provide a polished, smooth interface at the gingival border.

Subgingival Margins

Due to dental cavities, tooth inadequacies, or to conceal the tooth/restoration interface, restorative factors frequently influence the placement of the margin of the restoration behind the gingival tissue crest. The gingival attachment apparatus will be impacted by the margin of the restoration that is too deep beneath the gingival tissue crest, causing a persistent inflammatory reaction that is exacerbated by the patient's inability to properly clean the area. Gingival recession and bone loss originate from the body's attempt to generate space between the alveolar bone and the margin to allow for space for tissue reattachment. This is more prone to happen in regions where the alveolar bone is extremely narrowly encircling the tooth. Highly scalloped and thin gingiva is at the highest risk [6]. Considerations such as correct gingival third crown contour, proper buffing, rounded margins, sufficient connected gingival zone, and no deviation from BW should be made.

Rules for margin placement

The margins of the restoration could be positioned 0.5 mm below the gingival tissue crest if the sulcus probes 1.5 mm or less. It can be positioned in the sulcus at half its depth if the sulcus probes deeper than 1.5 mm. A gingivectomy could be done to extend the tooth and create a 1.5 mm sulcus if the sulcus is greater than 2 mm. If so, rule 1 can be applied to the patient's care.

Violation of biological width

**Figure 3:**
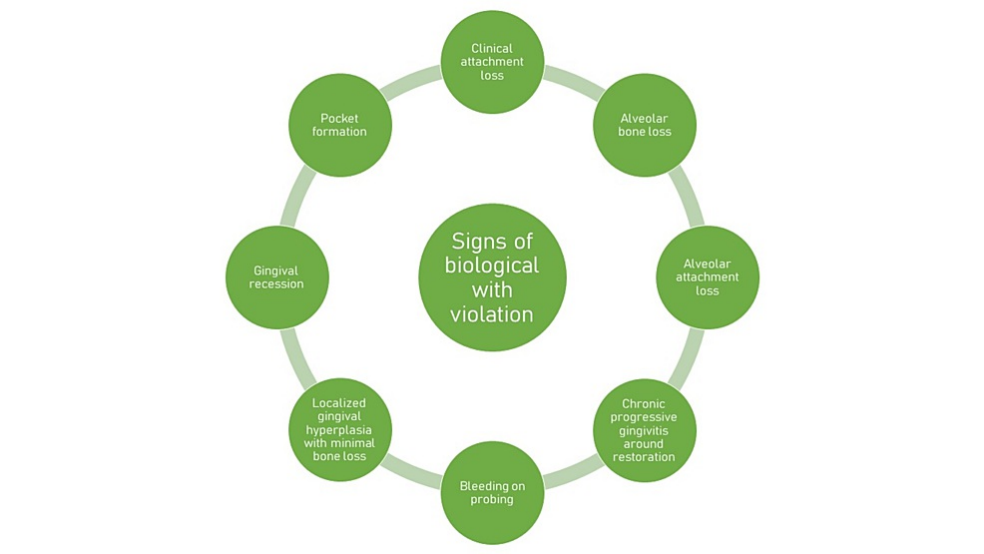
Signs of biological width violation

Subgingival restorations are notorious for being areas that retain plaque that are not accessible even with scaling instruments. Even though adequate and extensive supragingival plaque control measures are implemented, they seem to continue the process of accumulating plaque [24]. Histopathological examinations have revealed that gingival bevel crown margins are associated with compromised healing when compared to shoulder preparations. Subgingival restorations, when compared to supragingival restorations, are at an increased risk of bleeding along with gingival recession [25,26]. Teeth restored with subgingival restorations. which have narrower zones of keratinized gingiva, have higher gingival index scores than teeth with submarginal restorations and wider zones of keratinized gingiva. Before placing subgingival restorations, keratinized gingiva should be carefully examined [27]. Subgingival margins are found to be home to an increased number of spirochetes, fusiforms, rods, and filamentous bacteria (Figure 3) [28].

Correction of violation of biological width

In a situation where restoration of a tooth that has cracked or has become carious very near the alveolar crest, encroachment of biological breadth becomes of particular concern. Additionally, aesthetic considerations frequently call for burying margins of the restoration beneath the gingival margin, which forces them deep into the sulcus of the gingiva and violates biological width. Both intrapersonal and interpersonal heterogeneity exist within the biological width. Before making a final decision, each site and each patient must be assessed so as to respect the anatomy (Table 2) [29].

**Table 2:** Methods of biological width correction

Surgical crown lengthening	Orthodontic procedures
Gingivectomy: External bevel or internal bevel gingivectomy	Can be slow, rapid, forced tooth eruption with or without fibrotomy, supracrestal fibrotomy, and root planing (OEFRP)
Apical repositioned flap (ARF) surgery: with or without osseous reduction	

Surgical crown lengthening

The best treatment strategy for crown lengthening is chosen after conducting an analysis of each individual case with regard to the relationships between the crown and root of the alveolar bones (Table 3) [6,30].

**Table 3:** Indications, contraindications, and complications of surgical crown lengthening

Indications	Contraindications	Complications
Deficient clinical crown for retention because of deep and extensively large caries lesions, cemental/subgingival/root caries, or any type of tooth fracture, root perforation, or resorption within the cervical 1/3rd of the root in the teeth with suitable periodontal attachments	Deep carious or fractured tooth requiring an excessive amount of bone removal	Poor esthetics due to the presence of black triangles
Short or insufficient clinical crowns	Unjustified compromise of esthetics and/or of adjacent alveolar bone support	Root hypersensitivity
Excessive, unequal, and/or unesthetic gingival levels with respect to esthetics	Teeth that cannot be restored	Root resorption
Tooth with an imprudent amount of incisal or occlusal wear	Tooth depicting increased risk of furcation involvement	Transient tooth mobility
Teeth exhibiting weak interocclusal space for proper restorative procedures because of supraeruption		
Teeth exhibiting weak interocclusal space for proper restorative procedures because of supraeruption		
Tooth in need of hemisection or root resection		

Methods for surgical crown lengthening

Gingivectomy

Highly effective; however, it can only be utilized when there is hyperplasia or pseudo pocketing (> 3 mm of biological width) and a significant amount of keratinized tissue is present.

External Bevel Gingivectomy

External-bevel gingivectomy is a method of reducing an excessive pocket depth and/or exposing more coronal tooth structure when the attached gingiva is more than sufficient and there is no bone involvement [31].

Internal Bevel Gingivectomy

With or without the need to correct osseous abnormalities, minimizing the enormous depth of the pocket and exposing supplemental coronal tooth structure in the truancy of a sufficient zone of attached gingiva require surgical intervention. The flap must always be internally beveled in order to expose the supporting alveolar bone [5].

Apical Repositioned Flap (ARF) Surgery

Indicated when multiple teeth in a quadrant are in need of crown lengthening. This technique should never be used for the surgical crown lengthening of a single tooth, especially in the esthetic zone.

ARF Without Osseous Reduction

This is indicated in cases where the BW is more than 3 mm along with no adequate width amount of the attached gingiva.

ARF With Osseous Reduction

Indicated in cases where the BW is less than 3 mm, with no adequate width amount or zone of attached gingiva. Osteotomy followed by osteoplasty is done in order to expose the desired tooth length in a scalloped manner and to get the necessary contour of the overlying gingiva. It is a classic rule that at least 4 mm of sound tooth structure needs to be exposed, as the soft tissues will enlarge in a coronal direction to cover 2 to 3 mm of the root, leaving only 1 to 2 mm of supragingival sound tooth structure.

Orthodontic techniques

Slow Method

In this, low orthodontic forces are applied in a slowing erupting tooth. It brings the periodontium along with it. Extrusion of the tooth is performed until the bone level has achieved a coronal position when compared to the ideal position, i.e., the required area to address BW violation.

Rapid Method

In this, the tooth is allowed to erupt to the desired level for a number of weeks (while supracrestal fibrotomy is performed once a week to deliberately stop the bone and tissue from succeeding the tooth).

Forced Tooth Eruption Method

This method to treat "non-restorable" or previously "hopeless" teeth was first proposed by Heithersay and Ingber [32]. It is indicated when conventional crown lengthening through an ostectomy is not possible such as in the anterior region. When there is an insufficient crown-to-root ratio, insufficient occlusal clearance for the necessary quantity of eruption, and potential periodontal problems, forced tooth eruption is contraindicated [33].

Forced Eruption With Fiberotomy

This combines extrusion using orthodontic techniques and alienation of supracrestal fibers. The crestal bone and gingival margin are recovered in their pre-treatment locations if fibrotomy is done during the forced tooth eruption technique, leaving the tooth-gingiva interaction at neighboring teeth intact. During the forced tooth eruption phase, fiberotomies are performed once every 7 to 10 days [34].

Supracrestal Fiberotomy and Root Planing in Orthodontic Extrusion (OEFRP)

This* *corresponds to a flapless method for extending the crown following extrusion via orthodontic techniques. Throughout the entire extrusive orthodontic phase, the OEFRP technique must be performed every two weeks [35].

Future prospects

Although a considerable number of case reports are present, there is a need for clinical as well as laboratory-based research with respect to BW. Studies can be done on evaluating the BW in different populations and identifying which method of evaluation is the most accurate. This can be taken up as a comparative study. Studies focused on these aspects can ameliorate the current scientific literature.

## Conclusions

BW is vital to maintain the overall periodontal health, which in turn affects tooth health. Any violation due to improper restorative margins can lead to complications. In case of violations, procedures like surgical crown lengthening or orthodontic techniques can be employed to preserve the BW. Needless to say, BW is the natural seal protecting the periodontium and tooth, which should be preserved to maintain oral health.
